# IMPACTS OF ACCULTURATION ON HEALTH OF US OLDER CHINESE IMMIGRANTS

**DOI:** 10.1093/geroni/igad104.0485

**Published:** 2023-12-21

**Authors:** Yanping Jiang, Fengyan Tang, Bei Wu

**Affiliations:** Rutgers University, New Brunswick, New Jersey, United States; University of Pittsburgh, Pittsburgh, Pennsylvania, United States; New York University, New York City, New York, United States

## Abstract

Acculturation from Asian- to Western-oriented values and cultural practices is a key aspect of social experience for many Asian Americans. The growing body of literature on this topic shows mixed findings on health impacts of acculturation and is largely relied on cross-sectional studies, preventing to draw definitive conclusions on the health impact of acculturation. The study aimed to examine long-term effects of acculturation on mental, physical, and cognitive health in U.S. older Chinese immigrants. Data were drawn from 2,811 participants (aged from 60 to 104 years) from the Population Study of Chinese Elderly in Chicago study. Acculturation was measured using a 12-item Acculturation Scale assessing language use, ethnic social relations, and media use. Health outcomes included depression assessed using the Patient Health Questionnaire-9, activities of daily living (ADL) assessed using the adapted Katz ADL Scale, and global cognition indexed by a composite score of episodic memory, working memory, executive function, and mental status. Results from latent growth curve models showed that high levels of acculturation were not associated with depression at baseline but predicted a faster decline in depression during the 6-year follow-up. Similarly, acculturation was not related to ADL at baseline but predicted a slower decline over time. Contrarily, acculturation was associated with better global cognition at baseline but was not related to long-term changes in cognition. These results suggest the long-term benefits of acculturation on mental and physical health among U.S. older Chinese immigrants. The health advantages related to acculturation, however, seem not to generalize to cognitive function.

